# Sexual Mechanosensitivity: Age-Related Changes in the Innervation of the Human Prepuce

**DOI:** 10.3390/jcm14134730

**Published:** 2025-07-04

**Authors:** José A. Vega, Vincenzo Aiello, José Martín-Cruces, Iván Suazo, Ryan Jones, William Musa, Beatrix Szebeni-Varga, Olivia García-Suárez, Yolanda García-Mesa

**Affiliations:** 1 Departamento de Morfología y Biología Celular, Grupo SINPOS, Universidad de Oviedo, 33003 Oviedo, Spainivan.suazo@uautonoma.cl (I.S.); garciamyolada@uniovi.es (Y.G.-M.); 2 Facultad de Ciencias de la Salud, Universidad Autónoma de Chile, Providencia Área Metropolitana, Santiago 750012, Chile; 3 Foregen, Concord, CA 94520, USA; enzo@foregen.org (V.A.); ryan@foregen.org (R.J.); beatrix@foregen.org (B.S.-V.); 4 Instituto de Investigación Sanitaria del Principado de Asturias—ISPA, 33011 Oviedo, Spain

**Keywords:** human male prepuce, cutaneous end-organ complexes, Meissner corpuscles, mucous end-organ complexes, Krause-like corpuscles, mechanosensory innervations, age-dependent changes

## Abstract

**Background/Objectives**: The male prepuce that covers the glans penis is richly innervated by low-threshold mechanoreceptors, which form cutaneous end-organ complexes (Meissner, Pacinian and Ruffini corpuscles) and mucous end-organ complexes (especially Krause-like corpuscles). The mechanosensory inputs from these formations are the beginning for spinal reflexes that regulate movements of intercourse and erection and, therefore, are required for sexual function. The study was aimed at analyzing the age-dependent changes in prepuce innervation. **Methods**: Here we used immunohistochemistry to investigate whether the innervation of the male prepuce undergoes age-dependent changes, analyzing subjects aged 4 months to 61 years. **Results**: Abundant Meissner corpuscles and Krause-like corpuscles were regularly found whose morphology, size, and topography were variable and were not correlated with age; however, Ruffini’s and Pacinian corpuscles were scarcely observed. The earliest evidence of Meissner corpuscles was observed at 4 months, and thereafter they undergo significant age-dependent variations in density. Until the age of 20 years increases progressively, remains stable until 40 years, and then the density decreases. Meissner’s corpuscle index paralleled that of density. Regarding Kause-like corpuscles already resemble the skin of 4-month-old subjects and from the age of 3 years they can be identified at all ages. Its density significantly increased until 10 years and then remained stable. **Conclusions**: Present results state that the mechanosensory innervation of the human foreskin reaches its maximum value around the age of 20, remains stable during adulthood and decreases with maturity. These findings contribute to a more complete understanding of foreskin innervation and add to the scientific knowledge base surrounding the potential harm of removing a richly innervated structure.

## 1. Introduction

The male prepuce, also called foreskin, is a cutaneous-mucous structure that covers the glans penis; it is opened in the preputial meatus and fixed to the penis body at the preputial base [1,2]. Structurally, the prepuce consists of five layers that from the inside to the outside are mucosa, lamina propria, dartros muscle, dermis and epidermis. On the free edge of the prepuce, the cutaneous and mucosal epithelia continue although according to some authors the real transition occurs in the so-called ridged band [3]. For a detailed review of foreskin histology see [2].

The prepuce is richly innervated by sensory nerve fibers from the penile dorsal and ventral nerves [4] which are required for sexual function since they are the afferents for spinal reflexes that regulate movements of intercourse and erection [5]. The sensory nerve fibers end in the dermis and lamina propria of the foreskin forming cutaneous end-organ complexes (CEOCs) [6] and mucous end-organ complexes (MEOCs), respectively. CEOCs and MEOCs are classically known as sensory corpuscles. Most CEOCs and MEOCs, as well as the sensory complexes of the transitional zone are associated with sensory neurons which fall under the category of low-threshold mechanoreceptors (LTMRs). Characteristically the axons of LTMRs are fast conducting (ranging from 30 to 100 m/s, averaging ~40–60 m/s), have a large diameter, and are highly myelinated [6,7,8]. Different morphotypes of CEOCs, especially Meissner corpuscles and MEOCs have been reported in the mammalian prepuce, including men [1,3,9,10,11,12,13,14]. In addition to the axons of LTMRs, the prepuce is also innervated by nerve fibers belonging to the nociceptor category, i.e., type Aδ and C fibers, which form free nerve endings instead of CEOCs [6,7,8,15].

The terminology historically used to refer to the sensory nerve formations of the human foreskin is very complex (fibrils, papillary endings, and Vater-Pacini corpuscles, dermal bodies, Meissner’s corpuscles, Krause end bulbs, Dogiel’s bodies, Golgi-Mazzoni bodies, and genital corpuscles (see [12]). In the present study, the term Meissner’s corpuscle will be used as representative of the CEOCs (although the Pacini and Ruffini corpuscles also belong to this category) and the Krause-like corpuscle as a representative of the MEOCs (although other morphotypes that are difficult to classify can also be included).

On the other hand, according to some studies, the innervation of the foreskin is subject to age-dependent changes, and variations in the density of CEOCs and MEOCs have been observed at different ages. Özdemir-Sanci et al. (2024) [14] observed less CEOCs and free nerve endings in very young infants (0–3 years) compared with boys (6–11 years); and García-Mesa et al. (2021) [12] reported a tendency to decrease in the number of Meissner corpuscles after sexual maturity. These findings have been related to the acquisition of sexual maturity or decline. But they are surely also related to the aging of the foreskin tissues. In fact, human skin undergoes an aging process associated with deregulation of various cellular and inflammatory processes, as well as metabolic and hormonal causes [16]. In addition, aging is accompanied by a reduction in innervation density in the epidermis and dermis [17,18,19,20,21].

This study aimed to investigate the age-dependent changes in the density of the different types of CEOCs and MEOCs in the human male prepuce. Furthermore, we investigate whether the prepuce undergo age-related structural changes. The principal objective of this research was to contribute to clarify the neuroanatomical mechanisms underlying mechanosensitivity in the human foreskin.

## 2. Materials and Methods

### 2.1. Tissues

Prepuce samples were from 42 subjects (aged 4 months to 61 years) who underwent routine circumcision due to phimosis or redundant prepuce. Patients who showed signs of balanitis were excluded from the study. It should be emphasized that all tissues were obtained from surgical acts, by medical indication, and not by the desire of parents or tutors or by religious–cultural practices. The pieces were divided into five age groups based on age: 0–5 years (infants, n = 5), 6–10 years (pre-puberty, n = 6), 11–20 years (puberty, n = 17), 21–40 years (adult, n = 6), older than 41 years (mature, n = 8). The age groups were established solely on the basis of chronology and not on the basis of the subjects’ maturity or sexual activity, as the known data of the subjects included in the study were age and ethnicity (all were Caucasian). The samples were fixed in 4% buffered formaldehyde and routinely processed for paraffin embedding. In all cases, 1 cm of foreskin measured from the free edge, which included skin, mucosa and transition zone, was analyzed. Blocks were cut in 10 µm thick sections perpendicular to the skin/mucosa. Tissue samples were obtained in accordance with Spanish law (RD 1301/2006; Ley 14/2007; DR 1716/2011; Orden ECC 1414/2013).

The materials used in this study are a part of the histological collection of the Research Group SINPOS, Department of Morphology and Cell Biology of the University of Oviedo (Collections Section, Ref. C-0001627) created and authorized by the Ministry of Economy and Competitiveness of the Government of Spain on 30 November 2012.

### 2.2. Structural Study

Representative deparaffinized and rehydrated sections (3 per sample 200 µm apart) were processed for the performance of hematoxylin and eosin, and Masson’s trichrome staining techniques following routine procedures.

### 2.3. Immunohistochemistry

Deparaffinized and rehydrated sections were processed for indirect immunohistochemistry to detect S100 protein which label the terminal glial cells of CEOCs and MEOCs using the EnVision antibody complex detection kit (Dako, Copenhagen, Denmark) following the supplier’s instructions. Briefly, endogenous peroxidase activity was inhibited (3% H_2_O_2_ for 15 min), and non-specific binding was blocked (10% bovine serum albumin for 20 min). Sections were then incubated overnight at 4 °C with the primary antibodies. Thereafter, the sections were incubated with anti-rabbit EnVision system-labeled polymer (DakoCytomation) for 30 min. Finally, the slides were washed with buffer solution, and the immunoreaction was visualized with diaminobenzidine as a chromogen, washed, dehydrated, and mounted with Entellan (Merck, Dramstadt, Germany). To ascertain structural details, the sections were counterstained with Mayer’s hematoxylin. The primary antibodies against S100 protein used were a monoclonal antibody raised in mouse (Thermo Scientific, clone 4C4.9; Freemont, CA, USA) used at a dilution of 1:1000, and a polyclonal antibody raised in rabbit (Dako, Glostrup, Denmark) used diluted 1:2000. Both antibodies have been successfully used in the study of the innervation of the human foreskin [12].

For control purposes, some sections were processed in the same way as described above substituting the primary antibodies by non-immune rabbit or mouse or omitting the primary antibodies in the incubation. Under these conditions, no positive immunostaining was observed.

### 2.4. Quantitative Analyses of CEOCs and MEOCs

A quantitative analysis was carried out to determine the density of Meissner corpuscles at the different pre-established age groups using the methods proposed by Verendeev et al. (2015) [22], which has been described in detail in a previous study [21]. The Meissner index was determined according to Bhat et al. (2008) [9]. The densities of other CEOCs were not calculated because of their infrequent occurrence and irregular distribution in the dermis. Furthermore, the density of MEOCs was established counting the number in five randomly selected fields per section (2.5 mm^2^). For clarification: Density of CEOCs means the density of Meissner’s corpuscles per squared millimeter of skin (number of Meissner’s corpuscles/mm^2^) [22]; Index of CEOCs means the number of Meissner’s corpuscles with respect to the number or dermal papillae and was established in the same sections as the density [9]. Both measurements were standardized for the length of skin analyzed to compare between age groups.

Briefly, five sections, 200 µm apart, processed for S100 protein detection were scanned using an SCN400F scanner (Leica Biosystems™, Barcelona, Spain), and the scans were computed using SlidePath Gateway LAN software (Leica, Leica Biosystems™). Subsequently, Meissner’s corpuscles were identified and counted by two independent observers (YG-M and JAV). The average counts were corrected using the Abercrombie formula. The epidermal length (mm) of each section, and the average epidermal length was multiplied by the section thickness (mm) to calculate the surface area (mm^2^). Finally, the average number of Meissner’s corpuscles (N) was divided by the surface area (mm^2^) to calculate the density of Meissner’s corpuscles per square millimeter of skin (number of Meissner’s corpuscles/mm^2^). Subsequently, the average density was calculated from the individual densities for each pre-established age group.

To investigate the relationship between Meissner’s corpuscles and dermal papillae, the measurements were standardized according to the length of skin analyzed to compare between the groups. Statistical comparisons of Meissner’s corpuscle densities among the five predefined age groups were performed using the Kruskal–Wallis H test, due to the non-normal distribution of the data as determined by the Shapiro–Wilk test. When significant differences were observed, a post hoc Dunn’s test with Sidak correction was applied to identify specific group differences. Statistical significance was set at *p* < 0.05. All analyses were performed using MATLAB^®^ (MathWorks Inc., Natick, MA, USA).

## 3. Results

### 3.1. Age-Dependent Changes in the Structure of the Prepuce

**Figure 1:**
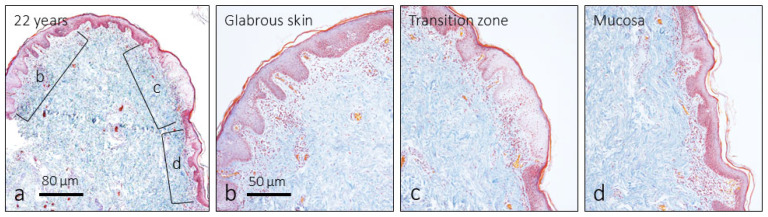
(**a**) Structure of the foreskin in the opening area. There is a difference between the skin on the outer side (**b**), mucous membrane on the inner side (**d**), and a transitional epithelium area between the two (**c**). Masson’s trichrome staining.

The structure of the human male foreskin of the samples examined using the Masson trichrome technique is that described by all the authors (Figure 1). The outer face is covered by glabrous skin, with the epidermis of keratinizing squamous epithelium (pigmented, without sebaceous or sweat glands) and the dermis (Figure 1b). The mucosa consists of a non-keratinizing squamous epithelium and a lamina propria (Figure 1d). The transition zone from the cutaneous to the mucous epithelium is marked by a non-keratinizing squamous epithelium higher than the others (Figure 1b); at this level, no greater presence of elastic fibers, or thickening of the dermis, was observed that would suggest a so-called ridged band. Both the dermis and the lamina propria are highly vascularized. The central part of the prepuce is occupied by smooth muscle cells of the dartos muscle invested with elastic fibers (Figure 1).

**Figure 2:**
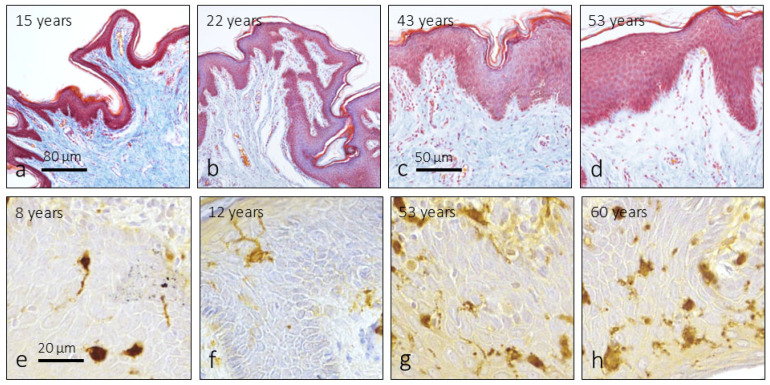
Structure of the foreskin at different ages (**a**–**d**). Masson’s trichrome staining. Cutaneous melanocytes in the foreskin display immunoreactivity for S100 protein at different ages (**e**–**h**).

The great irregularity shown in the cutaneous and mucosal epithelia in the histological sections makes it difficult to measure their thickness; there were also notable differences depending on the area analyzed in the same subject (Figure 2a–c). But, as a rule, no changes were observed in the structure of the foreskin as a function of age. One aspect found is that the melanocytes present in the epidermis (immunoreactive for the S100 protein) varied in density with age, and although there are large individual and intragroup variations, a tendency to increase with age was observed (Figure 2d–g).

### 3.2. Age-Related Changes in the Density of CEOCs and MEOCs in the Human Prepuce

**Figure 3:**
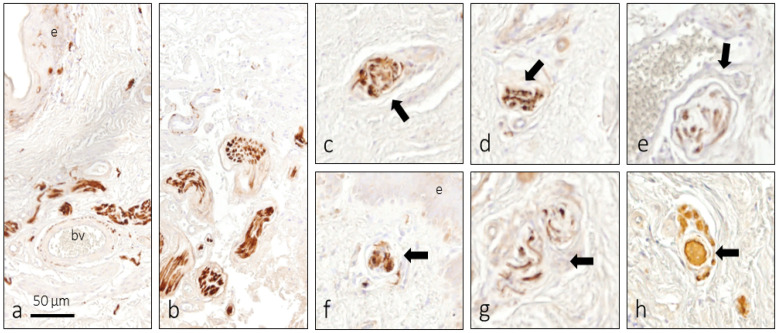
Nerves of the foreskin labeled by the immunoreactivity for S100 protein. In the dartros there are abundant nerve trunks and perivascular plexuses (**a**,**b**). In that same area, there are some cutaneous end-organ complexes, especially Ruffini ((**c**–**g**), arrows) and Pacini-like ((**h**), arrow). bv: Blood Vessels; e: Epidermis.

All the foreskin samples examined were densely innervated by nerve fibers of different calibers that form a network in the dermis, lamina propria and between the cells of the dartros muscle; from these nerves detaches branches that end up forming free nerve endings or associating with CEOCs and MEOCs. Furthermore, vegetative nerve fibers formed perivascular plexuses (Figure 3a,b). Nerve profiles were not observed in most dermal papillae, except where Meissner’s corpuscles were found. Epidermal free nerve endings were never observed.

In the deep layers of the prepuce closely related to the nerves some morphotypes of CEOCs were identified. As a rule, their density was very low, and they showed an irregular morphology. A few deeply located corpuscles displayed the morphology proper of Ruffini corpuscles (Figure 3c–g).

They were elongated, or rounded, depending on the section, and surrounded by a nearly developed capsule. Only in a single case one Pacinian corpuscle was identified (Figure 3h).

**Figure 4:**
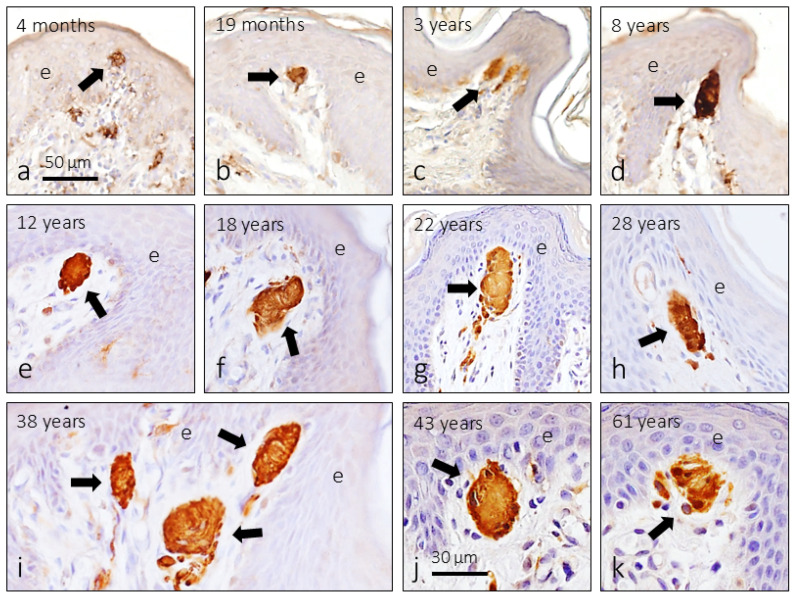
(**a**–**k**) Cutaneous end-organ complexes—Meissner’s corpuscles (arrows) located by immunoreactivity for the S100 protein in the foreskin of subjects of different ages. e: Epidermis. The scale bar is the same for all images.

CEOCs associated with the dermis were located beneath the epithelium and identified as Meissner’s corpuscles. They were observed both isolated and grouped in the cutaneous folds (Figure 4). The morphology, size, and distribution in the dermis of Meissner’s corpuscles were variable and were not correlated with age. On some occasions they had an ovoid appearance and were formed by stacks of lamellar cells; in others they were more rounded.

The earliest evidence of Meissner corpuscles was observed in a 4-month-old subject. These were small clusters of glial terminal cells at the bottom of the dermal papillae. Meissner’s corpuscles were observed at all subsequent ages. In terms of size, a progressive increase in size was observed until approximately 8–10 years of age; then it remains stable until the age of 38–40 and then reduces the size slightly.

**Figure 5:**
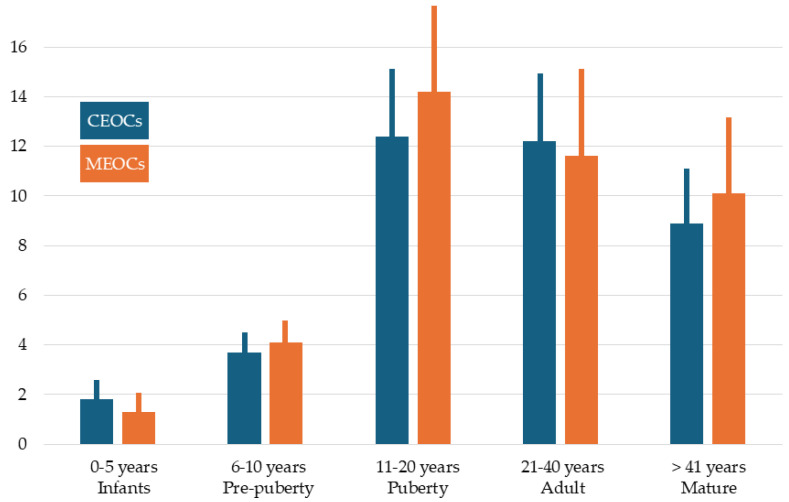
Graphical representation of the density and index of cutaneous end-organ complexes (CEOCs), and density of mucous end-organ complexes (MEOCs) in the human foreskin in the five age groups studied. Numerical data are in Table 1.

The density of CEOCs, which are represented in this study by Meissner’s corpuscles, undergoes notable age-dependent variations (Table 1 and Figure 5). Until the age of 20 years, the number of Meissner’s corpuscles increases progressively (multiplies by 10 from the first months), remains stable until the age of 40, and then decreases. Meissner’s corpuscle index paralleled that of density. Significant differences (*p* < 0.001) in both parameters were found between in infants and pre-puberty groups and those in the other three groups, while no significant differences in density or index of Meissner’s corpuscles were found between the puberty and mature groups.

The Kruskal–Wallis H test revealed statistically significant differences in Meissner’s corpuscle density among the five age groups (*p* < 0.001). Subsequent pairwise comparisons using Dunn’s post hoc test with Sidak correction indicated significant differences were found between: Group 1 (0–5 years) and Groups 4, and 5 (*p* < 0.01), Group 2 (6–10 years) and Groups 4 and 5 (*p* < 0.05), No significant differences were observed between Groups 4 (21–40 years) and 5 (>41 years) and Groups 3 (11–20 years) and 4. These results suggest a marked decrease in Meissner’s corpuscle density with increasing age, especially after puberty (Supplementary Materials).

**Figure 6:**
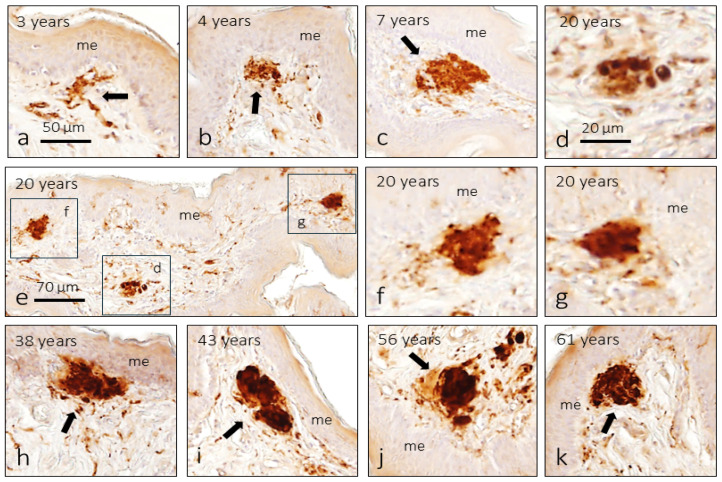
Mucous end-organ complexes—Krause’s corpuscles (arrows) located by immunoreactivity for the S100 protein in the foreskin of subjects of different ages. The image in (**d**) (is an enlargement of a detail of (**e**)) is a Ruffini-like CEOC localized in the dartros muscle and is not associated with epithelia. me: mucosae epithelium. The scale bar is the same for all images. The scale bar in a applies for (**a**–**c**) and (**h**–**k**); the scale bar in (**d**) applies for (**d**,**f**,**g**).

In relation to the lamina propria of the mucosa of the foreskin, sensory nerve formations (called MEOCs) were observed that do not have the morphology of Meissner’s corpuscles and that should be identified as Krause-like corpuscles. These are structures of very irregular morphology since the S100 protein-positive terminal glial cells are irregularly arranged within them (Figure 6). As with CEOCs, morphology and size are not related to age. These formations already resemble the skin of 4-month-old subjects, although they are very small, and from the age of 3, they can be identified at all ages.

In addition, and in general, its size apparently increased until the age of 6 and remains stable for the rest of life. On the other hand, the density of MEOCs increased until the age of 10 years and then remained stable. Significant differences (*p* < 0.001) were found between the infants and pre-puberty groups and those in the other three groups.

## 4. Discussion

The present research was designed to study age-related changes in the innervation of the human male foreskin. In addition, possible age-related changes in their structure were studied. The material used came from surgical acts, was structurally normal and only the age and ethnicity of the patients were known. In addition, it is considered that neither the cause of circumcision nor the surgical technique used to perform it influence the density of given CEOCs/MEOCs. On the other hand, we consider that the variations in the density of CEOCs/MEOCs should be attributed to age and not to possible factors, especially furnaces, since as far as we know no hormone receptors have been described in the cells that form the CEOCs/MEOCs.

The assessment of innervation was established using the S100 protein as a marker of nerves and sensory nerve formations, which is a selective marker of Schwann cells and glial terminal cells of CEOCs and MEOCs during development and in adults [8,23]. Generally, the terminal glial cells of CEOCs display intense S100P immunoreactivity regardless of their anatomical location [8].

We have established the density of CEOCs (Meissner’s corpuscles) related to skin innervation and MEOCs (a term coined by us for the first time in this study; Krause-like corpuscles) related to mucosal innervation. In addition, in the central area of the foreskin, in the dartos muscle, we have identified Ruffini-like and Pacini-like corpuscles (only one case observed), although these were not quantified due to their variability. These data are in good agreement with current knowledge about the morphotypes of CEOCs and MEOCs present in the human prepuce [1,3,9,10,11,12,13,14].

The sensory nerve formations associated with glabrous skin (CEOCs), mucous membranes (MEOCs) and the transition zones between them usually present a gradient from Meissner’s corpuscles in the outer cutaneous areas to glomerular corpuscles (Krausse’s corpuscle type) in the inner mucous areas. We have recently made observations on human lips [24] and clitoris [25]. As far as we know, this arrangement has never been studied in human foreskin.

From a functional point of view, all foreskin CEOCs and MEOCs serve different qualities of mechanosensitivity with rapidly adapting LTMRs, except for Ruffini’s corpuscles which are slowly adapting [7,26,27]. The prepuce receives somatosensorial innervation associated with erogenous sensations and sexual arousal [1]. Classically, it has been accepted that erogenous sensation is related to the predominance of CEOCs and MEOCs over free nerve endings. CEOCs detect different qualities of mechanosensitivity, while free nerve endings are associated with pain and to a much lesser extent with temperature and mechanical contact. However, this concept is not correct since C and Aδ fibers, which are the ones that form free nerve endings, are also related to mechanical stimuli [7,8,15].

On the other hand, in the present study, it has been observed that Meissner’s corpuscles begin their development in the postnatal period and acquire an appearance like that of adults [12] around 3–4 years of age. This temporal pattern is parallel to that of Meissner’s corpuscles of the fingers, although in them full development is acquired earlier, around 8–12 months. As far as we know, there are no data on the development of Krause corpuscles, but the results of this study suggest that it is like that of Meissner corpuscles.

The main objective of this work was to analyze possible age-related changes in the density of foreskin innervation. Our results show that the density, as well as the index, of Meissner corpuscles increases progressively until 20 years, stabilizes between 20 and 40 years, and then decreases until the age of 61. We do not know if they continue to reduce after 60 years as they do in digital skin [21]. As for the Krause-like corpuscles, the temporal evolution of their density was parallel to that of the Meissner corpuscles, although the reduction in the older subjects was smaller.

Thus, based on our results, it can be stated that the mechanosensory innervation of the human foreskin reaches its maximum value around the age of 20 (i.e., until puberty), remains stable during adulthood and decreases with maturity. The increase in CEOCs and MEOCs until adolescence might be related to the completion of maturation of the Meissner’s corpuscles or alternatively, it could be related to pubertal changes in the preputial skin. As for the reduction in older subjects, it cannot be certain whether this it is related to the cutaneous-mucosal microenvironment of the foreskin or to hormonal factors, although it is likely that it is both. In addition, it cannot be ruled out that it is a normal aging process of the peripheral somatosensory nervous system.

CEOCs and MEOCs are the places where mechanical stimuli are transformed into action potentials, and both CEOCs and MEOCs contain ion channels responsible for mechanotransduction [28], including Meissner’s corpuscles of the foreskin [12]. Mechanical stimuli play a key role in sexual arousal. However, whether the foreskin has a prominent role in sexual pleasure and orgasm is a matter of debate [29,30,31]. It has been reported that circumcision can decrease [32,33], or no change [34,35] penile sensitivity. A meta-analysis failed to demonstrate significant sexual alterations associated with circumcision [36]. However, although the effects of circumcision on penile sensitivity and sexual arousal vary between individuals but the prepuce is the most sensitive area of the penis [37]. In addition, given the complexity of the neurological and psychological mechanisms involved in sexual behavior, it is necessary to evaluate the long-term effects of all aspects of circumcision [38].

The findings of this study contribute to a more complete understanding of foreskin innervation at different stages of life, providing important context for assessing the sensory consequences of circumcision. By documenting age-related changes in mechanoreceptor density, this research adds to the scientific knowledge base surrounding the potential harm of removing a richly innervated structure in the genesis of the related mechanical inputs involved in sexual performance. In addition, these insights offer a foundational reference for future regenerative medicine efforts, where restoration of the foreskin’s sensory architecture will depend on detailed knowledge of its constitution at various ages.

## Figures and Tables

**Table 1:** Density and index of cutaneous end-organ complexes (CEOCs), represented by Meissner corpuscles (MC), and density of mucous end-organ complexes (MEOCs) in the human foreskin in the five age groups studied.

Age	Density of CEOCs (MC) Density of MEOCs	Index of CEOCs (MC)
0–5 years (n = 5). Infants	1.8 ± 0.4 1.3 ± 0.6	0.02 ± 0.001
6–10 years (n = 6). Pre-puberty	3.7 ± 1.2 4.1 ± 1.3	0.11 ± 0.01
11–20 years (n = 6). Puberty	12.4 ± 4.1 14.2 ± 5.1	0.22 ± 0.01
21–40 years (n = 6). Adult	12.2 ± 4.3 11.6 ± 5.3	0.25 ± 0.01
>41 years (n = 8). Mature	8.9 ± 3.3 10.1 ± 4.4	0.18 ± 0.01

n = number of cases. Values are expressed as mean ± standard deviation.

## Data Availability

The data that support the findings of this study are available from the corresponding author upon reasonable request.

## Supplementary Materials

The following supporting information can be downloaded at: https://www.mdpi.com/article/10.3390/jcm14134730/s1.
