# Supplementary material for: Sexual Mechanosensitivity: Age-Related Changes in the Innervation of the Human Prepuce
Source: J Clin Med. 2025 Jul 4;14(13):4730. doi: 10.3390/jcm14134730 (PMC12250474; doi:10.3390/jcm14134730)
Supplement: Supplementary file 1 [file jcm-14-04730-s001.zip › jcm-3677110-supplementary.pdf]

Post-hoc test results (Dunn with Sideak correction) after the Kruskal–Wallis analysis. The values are simulated. CI = confidence interval; the significance level was adjusted with Sidak's correction.

**Comparison between age groups**	**Estimated median difference**	**95% lower CI**	**95% Higher CI**	***P-Value* Corrected**	**Significant difference**
0–5 vs. 6–10 years	0.58	0.01	1.15	0.043	Yes
0–5 vs. 11–20 years	2.05	1.54	2.56	<0.001	Yes
0–5 vs. 21–40 years	2.40	1.80	3.00	<0.001	Yes
0–5 vs. >41 years	2.65	2.10	3.20	<0.001	Yes
6–10 vs. 11–20 years	1.47	0.96	1.98	<0.001	Yes
6–10 vs. 21–40 years	1.82	1.20	2.44	<0.001	Yes
6–10 vs. >41 years	2.07	1.48	2.66	<0.001	Yes
11–20 vs. 21–40 years	0.35	-0.07	0.77	0.091	No
11–20 vs. >41 years	0.60	0.18	1.02	0.006	Yes
21–40 vs. >41 years	0.25	-0.18	0.68	0.210	No
